# Supplementary material for: Neurorehabilitation of the upper extremity – immersive virtual reality vs. electromechanically assisted training. A comparative study
Source: Front Neurol. 2023 Dec 21;14:1290637. doi: 10.3389/fneur.2023.1290637 (PMC10768030; doi:10.3389/fneur.2023.1290637)
Supplement: Supplementary file 1 [file Data_Sheet_1.docx]

***Supplemental Material***


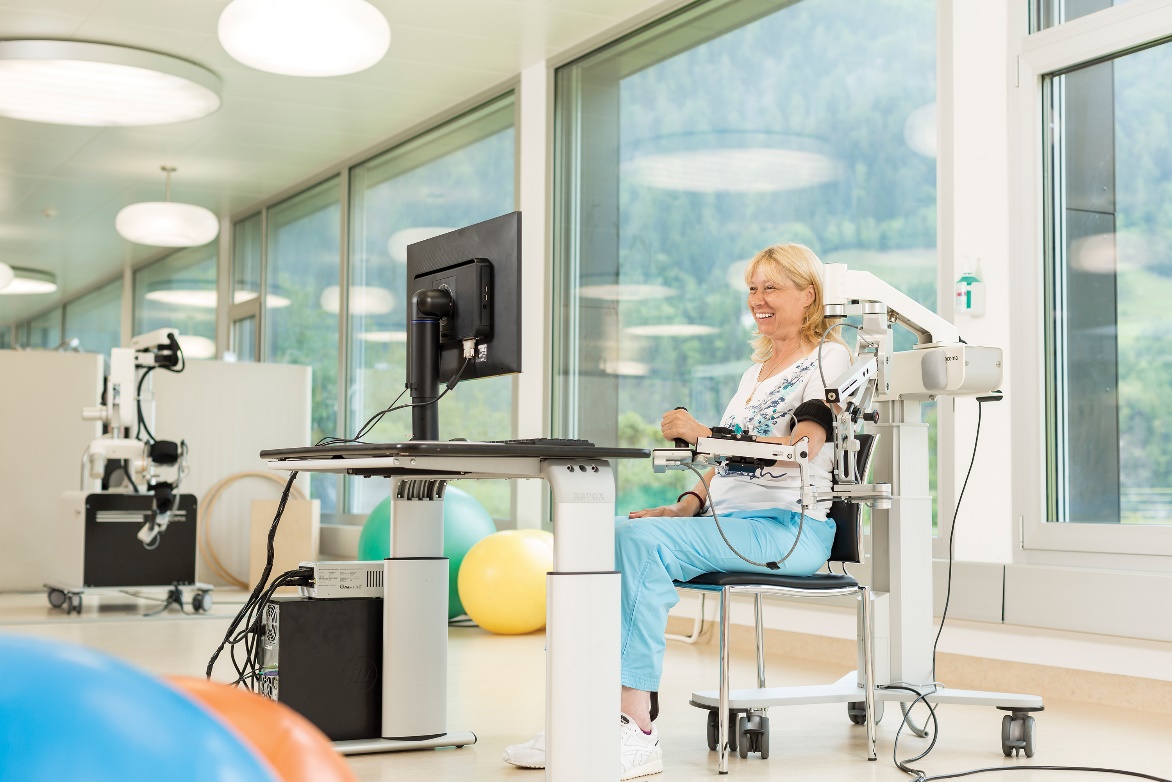


**Figure 1:** Image of the ARMEOSpring®. Image credit – Hocoma, www.hocoma.com


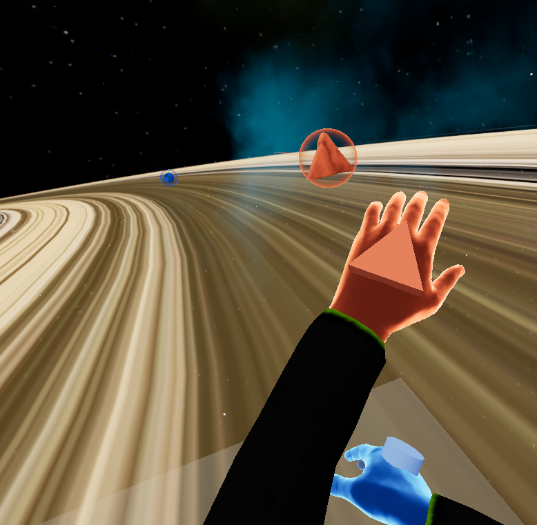

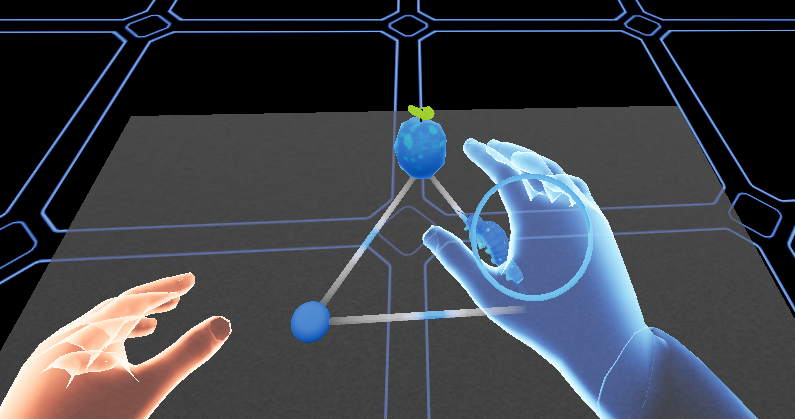


**Figure 2:** The iVR-based unilateral arm training. On the left, a caterpillar has to be guided along a pre-defined path towards a fruit. On the right, moving meteorites have to be caught by “touching” them with the affected hand.
